# Effects of azithromycin on alleviating airway inflammation in asthmatic mice by regulating airway microbiota and metabolites

**DOI:** 10.1128/spectrum.02217-24

**Published:** 2025-02-11

**Authors:** DanHui Huang, Lingyan Xie, Tingyue Luo, Lishan Lin, QianNan Ren, Zhaojin Zeng, Haohua Huang, Hua Liao, XiaoDan Chang, Yuehua Chen, Haijin Zhao, Shaoxi Cai, Hangming Dong

**Affiliations:** 1 Chronic Airways Diseases Laboratory, Department of Respiratory and Critical Care Medicine, Nanfang Hospital, Southern Medical University, Guangzhou, China; 2 Department of Radiation Oncology, Nanfang Hospital, Southern Medical University, Guangzhou, Guangdong, China; FIND, Geneva, Switzerland

**Keywords:** asthma, azithromycin, airway microbiota, sphingomyelin metabolism

## Abstract

Asthma is a chronic respiratory disease with increasing global prevalence, often linked to disrupted airway microbiota. Azithromycin has shown promise in asthma treatment, but whether its effect is owing to its antimicrobial capacity remains largely unknown. A house dust mite (HDM)-induced asthmatic mouse model was used to evaluate the effects of azithromycin on airway inflammation and microbiota. Mice were divided into control, HDM-induced asthma, HDM + azithromycin, and azithromycin-alone groups. Airway microbiota was analyzed using 16S rRNA sequencing, and metabolomic profiles were assessed via liquid chromatography–tandem mass spectrometry. Azithromycin alleviated type 2 airway inflammation in HDM-induced asthma, restoring microbiota diversity by modulating specific genera, including *Streptococcus*, *Staphylococcus*, *Ruminococcus*, *Coprococcus*, *Bifidobacterium,* etc. Combination analysis with metabolomics revealed that azithromycin significantly regulated airway microbiota-associated sphingomyelin metabolism. Azithromycin’s therapeutic effects in asthma are associated with its ability to regulate airway microbiota and its associated sphingomyelin metabolism, highlighting the potential for microbiota-targeted therapies in asthma.

**IMPORTANCE:**

Asthma, a prevalent chronic respiratory condition, poses a significant global health challenge due to its increasing prevalence and associated morbidity. The role of airway microbiota in asthma pathogenesis is gaining attention, with evidence suggesting that disruptions in this microbial community contribute to disease severity. Our study investigates the impact of azithromycin, a macrolide antibiotic, on airway inflammation and microbiota in a mouse model of asthma. The findings reveal that azithromycin not only alleviates airway inflammation but also restores microbiota diversity and modulates microbiota-associated sphingomyelin metabolism. This research underscores the potential of microbiota-targeted therapies in asthma management, offering a novel therapeutic strategy that could improve patient outcomes and reduce the healthcare burden associated with asthma.

## INTRODUCTION

Asthma is the most prevalent chronic respiratory disease worldwide, with an increasing number of cases over the years (1). While mortality rates have decreased, a significant proportion of asthma patients do not achieve optimal control, leading to higher healthcare utilization and economic costs (2). Recent research has highlighted the significant role of the microbiome in the pathogenesis, phenotype, and treatment of asthma (3). Multiple studies have found that children living in farm settings have a lower risk of allergic diseases than children in urban settings (4). The lower the bacterial and fungal diversity in house dust, the higher the rate of asthma in the pediatric population (5), suggesting that the changes in the structure of the microbiota in the environment may be closely related to the occurrence of asthma.

In the past, scholars believed that the lungs of healthy people were sterile, while studies have found that there are rich bacterial groups in different locations of the airways of healthy people (6). The airway microbiota in the normal population has an influence on the immunity of the whole body, and its equilibrium state represents the cumulative effect on the local and overall innate and adaptive immune processes affected by the microorganisms and their metabolite components (7, 8). If the healthy microbial colonization process is disturbed, the airway microbiota will become an important risk factor for the development of many respiratory diseases (9). Asthmatic lungs show a distinct microbiota composition, with a higher prevalence of *Proteobacteria*, compared to healthy lungs, which are dominated by *Bacteroidetes* (10, 11). Reduced diversity in the lung microbiota is associated with asthma (12). The microbiome’s impact on asthma is multifaceted. A study reports that *Moraxella*, *Streptococcus*, and *Haemophilus* increase the risk of childhood rhinovirus infection, which in turn increases the risk of asthma occurrence and acute exacerbation (13). Moreover, airway microbiota are closely related to asthma airway hyper-responsiveness (14) and airway inflammatory phenotype (15).

According to the Global Initiative for Asthma guidelines, azithromycin (AZM), a macrolide antibiotic, has been studied for its potential benefits in managing severe asthma patients who still have persistent symptoms and require medium and high doses of ICS/LABA, regardless of the inflammation phenotype (16). The AMAZES study from Australia reports that azithromycin reduces the rates of exacerbations and improves asthma-related quality of life in asthma patients (17). A meta-analysis concluded that azithromycin administration reduced exacerbations in severe asthma patients with both eosinophilic and non-eosinophilic phenotypes (18). The mechanisms by which azithromycin exerts its effects in regulating airway inflammation in asthma are not fully understood. One hypothesis is that azithromycin may directly affect host immune cells and signaling pathways, independent of their antimicrobial properties (19). For example, azithromycin modulates the function of human monocyte-derived dendritic cells and CD4+ T cells, potentially benefiting patients with inflammatory disorders (20). However, since increasing evidence suggests that airway microbiota has a close link with airway inflammation (21, 22), could azithromycin exert its anti-inflammatory effects by regulating airway microbiota in asthma patients? A double-blind, multicenter, randomized clinical trial found that azithromycin reduced the airway microbiota diversity and reduced lung inflammatory factor levels in 20 smokers with emphysema (23). A recent study reported that long-term usage of azithromycin reduced the abundance of sputum *Haemophilus influenzae* in severe asthma patients (24). The above studies suggest that azithromycin has a regulatory effect on the airway microbial community, and this effect may be involved in immune regulation and anti-inflammatory responses.

To investigate the hypothesis that azithromycin might modify the composition of the airway microbiome, potentially fostering a more balanced microbial ecosystem ,which subsequently modulates immune responses and alleviates inflammation, we embarked on an exploration of the relationship between azithromycin and its impact on the airway microbiota. Our research revealed that azithromycin was effective in restoring disturbances within the airway microbiota. Metabolomic analysis indicated that sphingomyelin metabolism was notably altered and correlated with changes in the airway microbiota. Furthermore, sphingomyelin exhibited a protective role in alleviating airway inflammation in asthmatic mice.

## MATERIALS AND METHODS

### Reagents

Azithromycin was purchased from Zithromax, and Sphingomyelin from Medchemexpress (HY 113498). Mouse ELISA kit was purchased from Abclonal, including IL-4 (RK 00036), IL-5 (RK 00037), and IL-13 (RK 00107).

### Animal and experiment design

BALB/c mice (male, 6 weeks old, 18–22 g) were purchased from Guangdong Medical Laboratory Animal Center with the approval of the Laboratory Animal Ethics Committee of Southern Medical University. House dust mite (HDM)-induced asthmatic mouse model: 28 BALB/c mice were randomly divided into four groups: (i) control group (CN group), (ii) HDM group, (iii) HDM + azithromycin group (HDMAZM group), and (iv) azithromycin group (AZM group). The HDM-induced asthmatic mouse model was constructed based on the previous method (25). To validate SM effect on asthma: 28 BALB/c mice were randomly divided into four groups: (i) control group (CN group), (ii) HDM group, (iii) sphingomyelin group (SM group), and (iv) HDM + sphingomyelin group (HDMSM group). For the SM group, 15 µL saline containing 10 µg of sphingomyelin was administrated intranasally daily. The same volume of saline was used as a control.

### Collection of mice feces and bronchoalveolar lavage fluid

Mouse fecal samples were collected using the tail-lifting method. Mice were stimulated to defecate, and the feces were collected using a 1.5 mL sterile centrifuge tube, with approximately 1 g of sample (about one to three pellets), and no less than 0.2 g. The collected samples were immediately stored at −80°C for preservation. Bronchoalveolar lavage fluid (BALF) samples were collected by flushing the lungs three times with 2 mL PBS using a 1 mL syringe inserted into a cannula. Cell suspension samples were prepared for total cell count and for blinded assessment of eosinophil cell percentages with HE stain.

### 16s rRNA sequencing analysis

BALF samples and fecal samples were transferred to Noomi Metabolism for 16S rRNA sequencing following the protocol. Libraries were prepared with the NEBNext Ultra II DNA Library Prep Kit following the manufacturer’s protocol. Quality assessment was performed using the Qubit 2.0 Fluorometer and Agilent Bioanalyzer 2100.

Sequencing was conducted on the Illumina NovaSeq platform generating 250 bp paired-end reads. Raw data were filtered to remove adapter contamination and low-quality reads. Amplicon sequence variants were generated using QIIME2 (26) and DADA2 (27), with taxonomy assigned via the SILVA database v138 using the naive Bayesian classifier in QIIME2.

### Microbiota analysis

Microbiota community structure was analyzed using α diversity levels, as well as β diversity levels. α diversity was assessed using Simpson and Shannon indices. Beta diversity was estimated using unweighted UniFrac and weighted UniFrac distances and visualized by principal coordinate analysis (PCoA). UPGMA Cluster heatmap based on Euclidean distance was used to show genera with similar distribution among groups. PICRUSt2 (28) was used to predict the functional profiles of microbial communities based on 16S rRNA sequences.

### Non-targeted metabolomics by LC-MS/MS sequencing analysis

BALF samples were transferred to Zhongke New Life Company for non-targeted metabolomics analysis following the protocol. Liquid chromatography–tandem mass spectrometry (LC-MS/MS) analyses were performed using a Vanquish UHPLC system coupled with an Orbitrap Q ExactiveTM HF mass spectrometer (both Thermo Fisher Scientific, Germany) at Novogene Co., Ltd. (Beijing, China). Raw data were processed using Compound Discoverer 3.1 (Thermo Fisher Scientific) with parameters set as follows: retention time tolerance of 0.2 min, actual mass tolerance of 5 ppm, signal intensity tolerance of 30%, signal-to-noise ratio of 3, and minimum intensity. Peak intensities were normalized to total spectral intensity, and molecular formulas were predicted based on additive ions, molecular ion peaks, and fragment ions. Peaks were matched against the mzCloud, mzVault, and MassList databases for accurate qualitative and relative quantitative results.

### Metabolomics data analysis

Metabolites were annotated using the KEGG, HMDB, and LIPIDMaps databases. Orthogonal partial least squares discriminant analysis (OPLS-DA) was performed to model the differences between groups. To analyze the expression patterns of all qualitative metabolites in each group and characterize the changing trend of metabolite expression levels. The fuzzy c-means (FCM) algorithm was employed for analysis. Mann-Whitney *U* test was used to calculate statistical significance (*P*-value) to identify differential metabolites.

### Protein extraction and analysis of lung tissues

Lysate was prepared in a proportion of 5:1 (100 mg of right lung tissue was added to 500 µL of lysate) and 1 mM PMSF. The supernatant was obtained after grinding and centrifugation (12,000 rpm, 20 min) and stored at 4°C. Measurement of cytokines IL-4, IL-5, and IL-13 was completed within 1 week according to the manufacturer’s instructions. Lung sections were stained with hematoxylin and eosin (H & E) to evaluate the degree of inflammatory cell infiltration around the bronchus and Periodic Acid Schiff stain (PAS) to assess airway mucus secretion.

### Statistical analyses

All experimental statistics were obtained using GraphPad Prism 7. The independent sample *t*-test or one-way analysis of variance was used for normally distributed numerical variables, while skewed numerical variables were tested using the Kruskal-Wallis non-parametric test or the Dunn’s *post hoc* test. Differences in various detection indexes of metabolites and cytokines were analyzed according to the Mann-Whitney *U* test and the Kruskal-Wallis test. The calculation of the correlation between the genus and metabolites usually uses the Spearman correlation coefficient, *P* value < 0. 05 is regarded as statistically significant; the correlation coefficient *r* < 0 represents a negative relationship; *r* > 0 represents a positive correlation; | r | size represents the correlation size; and the value range is (−1, 1).

## RESULTS

### Azithromycin alleviates airway inflammation in HDM-induced asthma model

**Fig 1:**
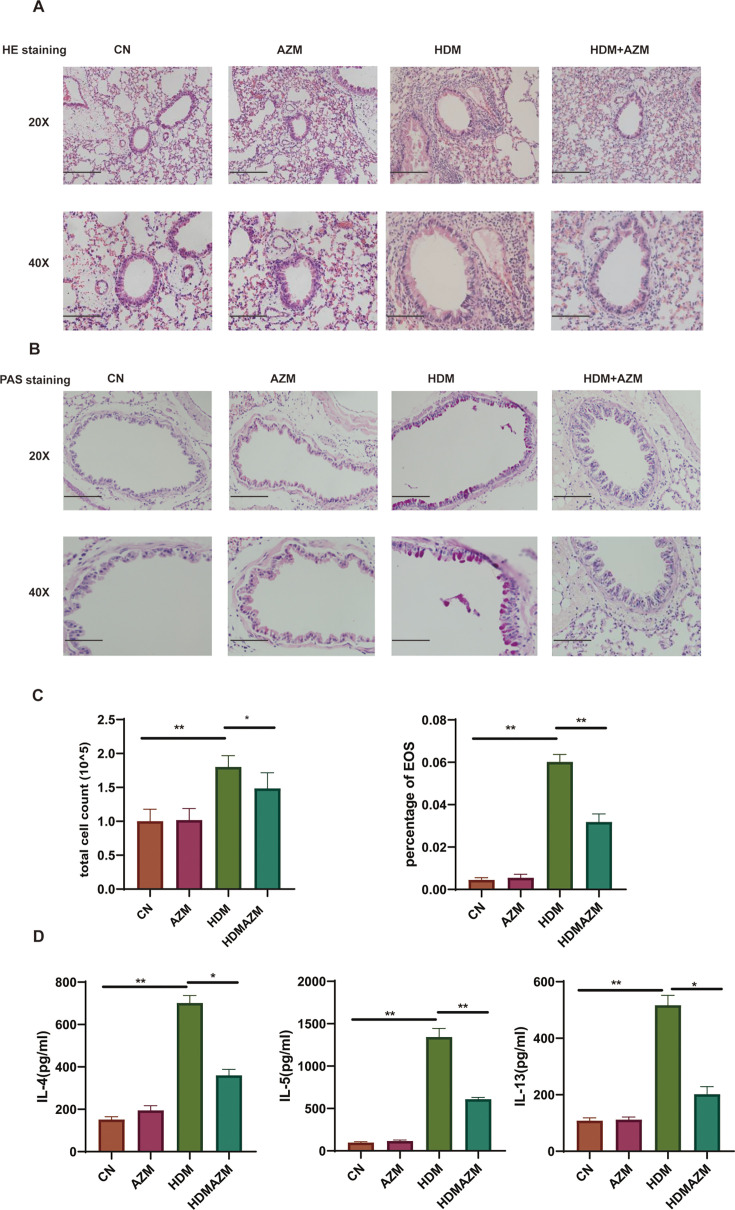
Azithromycin alleviates airway inflammation in HDM-induced asthma mice model. (**A**) Representative H & E-stained lung tissue sections from different groups. Magnification, 20× (the upper panel, scale bar = 200 µm) and 40× (the lower panel, scale bar = 100 µm). (**B**) Representative PAS-stained lung tissue sections from different groups. Magnification, 20× (the upper panel, scale bar = 200 µm) and 40× (the lower panel, scale bar = 100 µm). (**C**) BALF total cell count and BALF eosinophilia percentage among groups. *P < 0.05 and **P < 0.01. (**D**) BALF IL-4, IL-5, and IL-13 levels among groups. Data are expressed as mean ± standard deviation. *P < 0.05 and **P < 0.01.

Lung histopathology sections for HE staining in four groups are shown in Fig. 1A. Compared with the control group, HDM asthmatic mice had obvious inflammatory cell infiltration. Importantly, when HDM asthmatic mice were administered with AZM, airway inflammatory cell infiltration was significantly reduced. AZM treatment alone will not cause significant airway inflammation. In addition, mucus secretions in airway epithelial cells were evaluated in the four groups (Fig. 1B). Similarly, HMD-induced asthmatic mice had significantly increased mucus secretion, which was reduced by AZM administration. Measurement of BALF inflammatory cell counts (Fig. 1C) and T2 cytokines (Fig. 1D) in four groups was conducted. The results showed that compared with the control group, the HDM group had observably increased BALF total cell counts, eosinophil (EOS) percentage, and IL-4, IL-5, and IL-13 levels. In conclusion, consistent with the previous reports (29), AZM significantly reduced type 2 airway inflammation and mucus secretion in HDM asthmatic mice.

### Azithromycin restored airway microbiota dysbiosis in asthmatic mice

Commensal microbiota may affect asthma progression. We speculate that the protective effect of AZM on asthma is associated with its antimicrobial function. BALF and fecal samples were collected for 16S rRNA sequencing. α diversity was used to reflect the richness and uniformity of species within the microbial community and is characterized by the Shannon index and the Simpson index. To test the relationship between asthmatic airway inflammation and microbiota in the airway and intestines, Pearson analysis was conducted. The results showed that IL-5 level and EOS percentage were significantly associated with the Simpson and Shannon index of BALF microbiota, rather than fecal microbiota (Fig. S1), indicating that airway microbiota may have a closer connection with asthma progression. Therefore, we focus on airway microbiota for further analysis.

**Fig 2:**
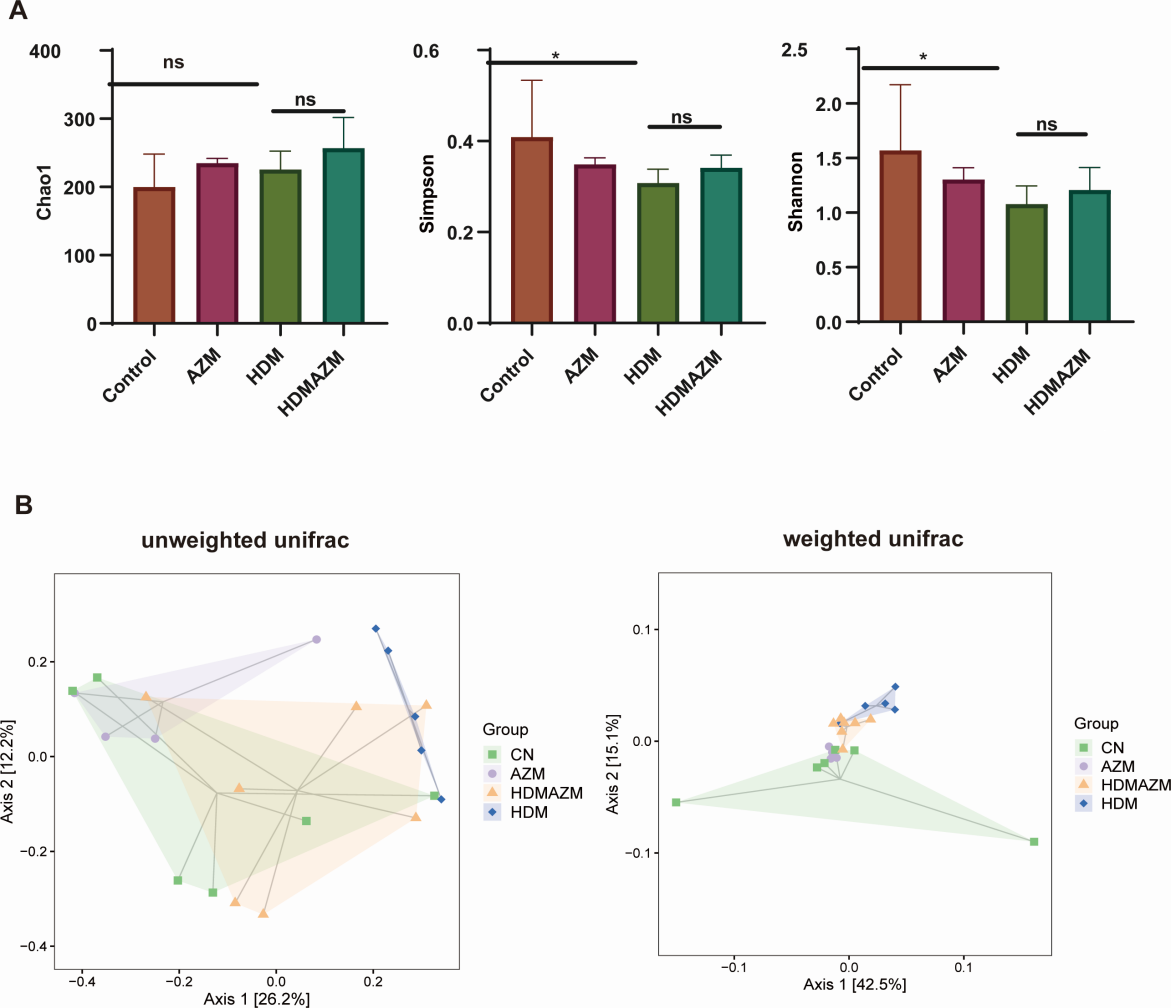
Azithromycin partially restores airway microbiota dysbiosis in HDM-induced asthma mice model. (**A**) α-diversity index (Chao1, Simpson, and Shannon) of BALF microbiota among groups. *P < 0.05. ns, nonsignificant. (**B**) PCoA based on weighted UniFrac and unweighted UniFrac distance among groups.

Taxonomy composition of the four groups is shown in Fig. S1. The top three phyla of the four groups were *Proteobacteria*, *Actinobacteria*, and *Firmicutes*, while the top five genera of the four groups were *Burkholderia*, *Acinetobacter*, *Caulobacteraceae*, *Prauserella*, and *Mycoplana* (Fig. S2). The α diversity of BALF microbiota in four groups was generally different (Fig. 2A). The HDM group exhibited a significant reduction in both the Simpson index (Mann-Whitney *U* test, *P* = 0.032) and Shannon index (Mann-Whitney *U* test, *P* = 0.042) when compared to the CN group. The HDMAZM group demonstrated that the administration of AZM has the potential to restore α diversity, although the results did not achieve statistical significance. In comparison to the HDM group, the HDMANZ group showed an upward trend in both the Simpson index (Mann-Whitney *U* test, *P* = 0.351) and Shannon index (Mann-Whitney U test, *P* = 0.252) compared to the HDM group. β diversity was characterized by PCoA based on unweighted UniFrac distance, and weighted UniFrac distance showed a significantly different community structure in HDM (Fig. 2B) compared with CN (PERMANOVA test, *P*＜0.01 for both unweighted UniFrac distance and weighted UniFrac distance). Importantly, community structure difference was partially eliminated via AZM administration, as PCoA plots revealed that, in comparison to the HDM group, the HDMAZM group exhibited a significantly different community structure (PERMANOVA test, *P*＜0.01 for both unweighted UniFrac distance and weighted UniFrac distance) and was more closer to the CN group.

**Fig 3:**
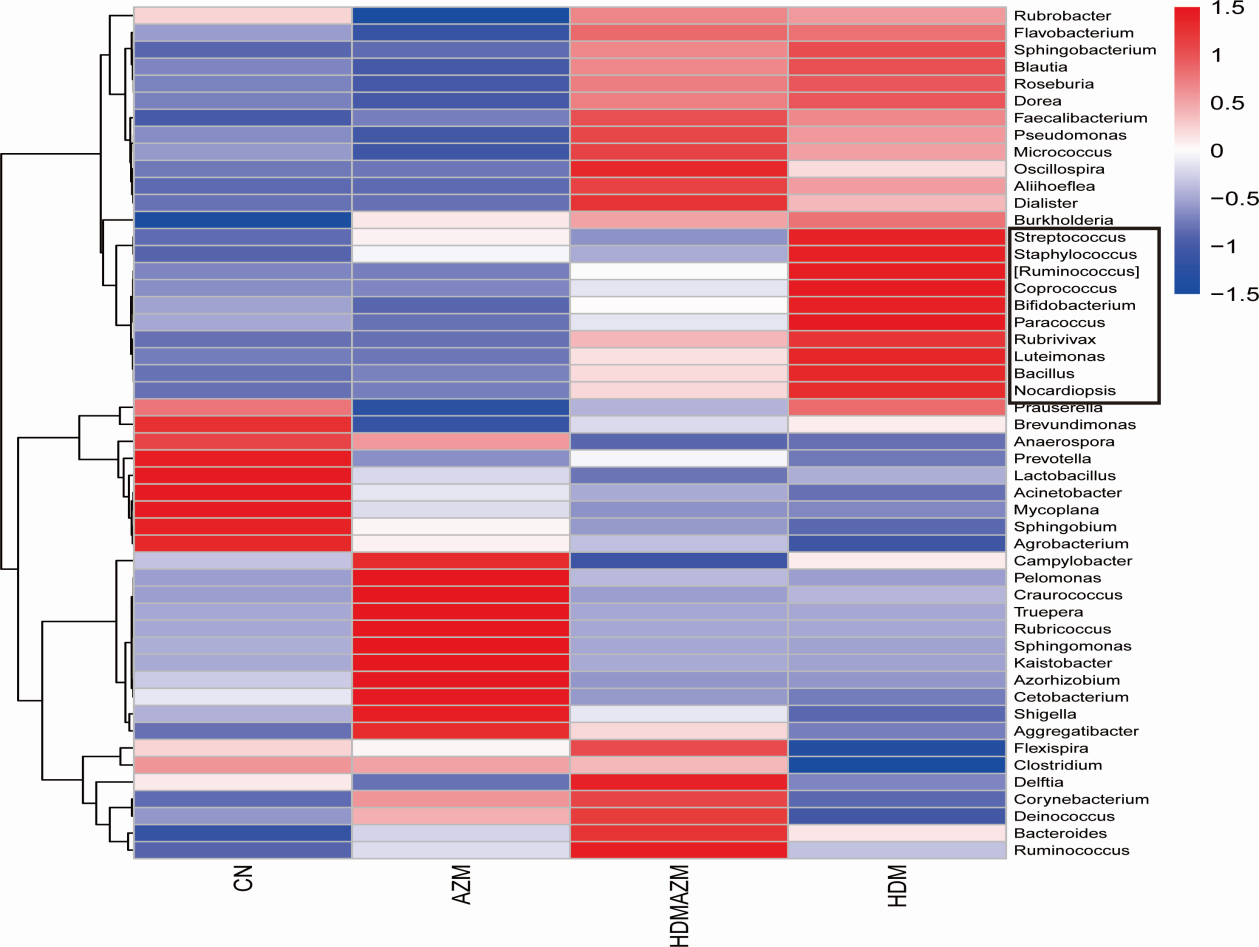
UPGMA cluster heatmap based on Euclidean distance showed the top 50 genera with similar distribution among groups. *Streptococcus*, *Staphylococcus*, *Ruminococcus*, *Coprococcus*, *Bifidobacterium*, *Paracoccus*, *Rubrivivax*, *Luteimonas*, *Bacillus*, and *Nocardiopsis* are found with increased trend in HDM asthmatic mice and can be decreased by AZM treatment.

Given that AZM showed potential in mitigating airway microbiota imbalance in asthmatic mice, we explored further to identify the key genera that underwent significant alterations in asthma and were subsequently restored upon AZM administration. Clustered heatmap (Fig. 3) shows the relative abundance distribution trends of the top 50 genera in each group, and genera with similar change pattern were clustered together. Interestingly, *Streptococcus*, *Staphylococcus*, *Ruminococcus*, *Coprococcus*, *Bifidobacterium*, *Paracoccus*, *Rubrivivax*, *Luteimonas*, *Bacillus*, and *Nocardiopsis* are a group of genera found with an increased trend in asthmatic mice and can be decreased by AZM treatment. Further analysis demonstrated that besides *Staphylococcus* and *Coprococcus,* other eight genera were statistically significantly increased in the HDM group and partially eliminated by AZM administration (Fig. S3). These eight genera might be potential bacterial candidates associated with AZM treatment effect for asthma.

In conclusion, our results showed that airway microbiota dysbiosis existed in asthmatic mice, and AZM administration can partially restore airway microbiota balance by regulating microbiota diversity and specific genera.

### Azithromycin regulates multiple lung metabolites in asthmatic mice

**Fig 4:**
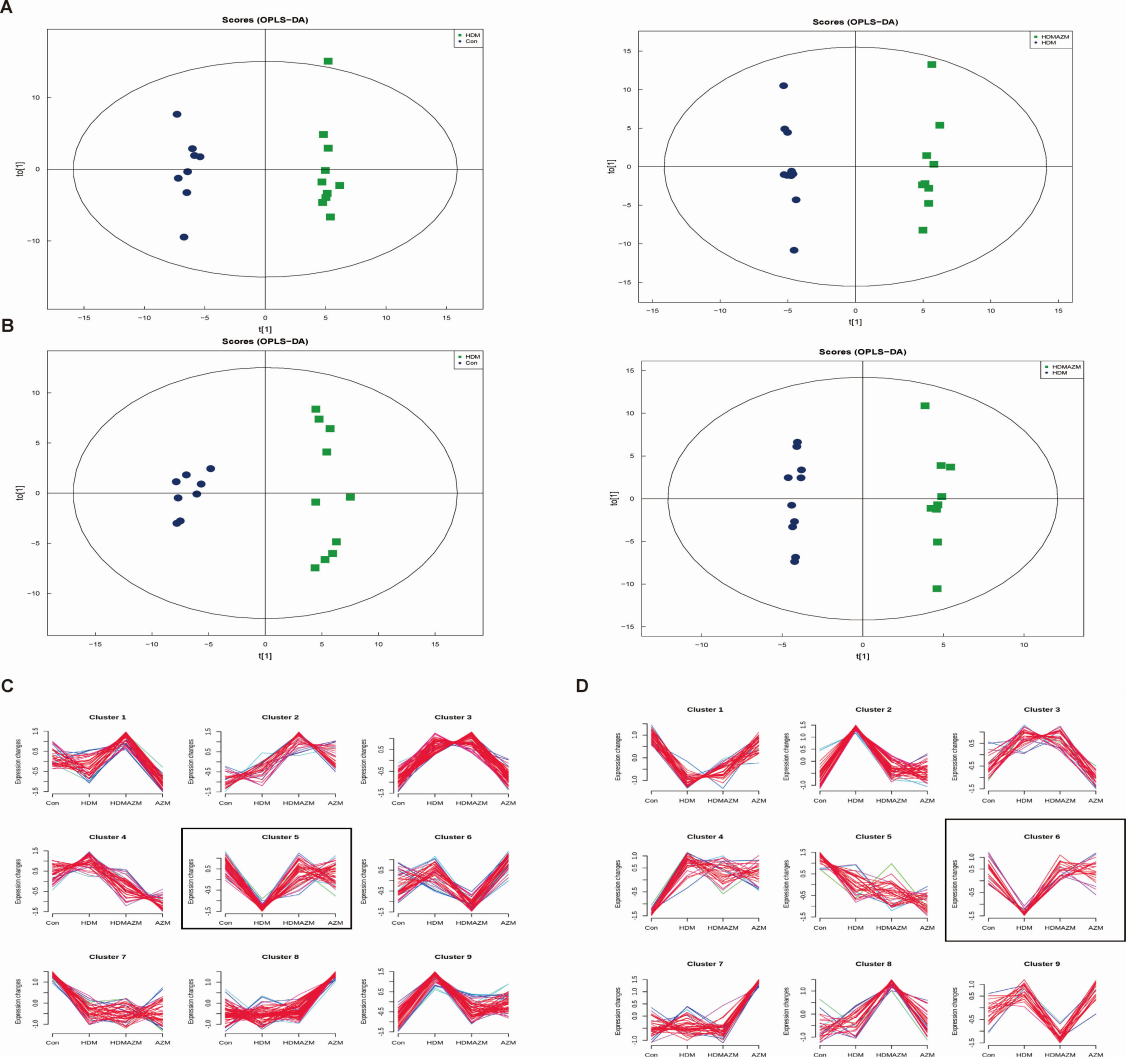
Metabolic profile among groups. (**A**) OPLS-DA of metabolites in positive ion mode identifies significant metabolic profile between the HDM and CN groups, and between the HDM and HDMAZM groups. (**B**) OPLS-DA of metabolites in negative ion mode identifies significant metabolic profile between the HDM and CN groups, and between the HDM and HDMAZM groups. (**C**) Metabolites in positive ion mode were clustered with different expression modules according to their expression trends via fuzzy c-means analysis. Cluster 5 is a group of metabolites with obvious change in asthmatic mice and would be restored by AZM administration. (**D**) Metabolites in negative ion mode were clustered with different expression modules according to their expression trends via fuzzy c-means analysis. Cluster 6 is a group of metabolites with obvious change in asthmatic mice and would be restored by AZM administration.

Microbiota produce a wide range of metabolites that influence host physiology, including short-chain fatty acids and bile acids, which are involved in energy metabolism, immune modulation, and cellular processes (30). In order to identify metabolomic patterns and microbiota-associated metabolites, LC-MS/MS analyses were performed. Totally, 707 metabolites were quantified and identified in the positive and negative ion modes, mainly including lipids and lipid molecules, organic acids and derivatives, benzenoids, organic oxygen compounds, and organic nitrogen compounds (Fig. S4). OPLS-DA was conducted to identify metabolomic patterns among groups. The results showed that metabolite profiles differed significantly between the HDM and CN groups, and between the HDM and HDMAZM groups (Fig. 4A and B).

Meanwhile, by using FCM, we divided all metabolites into different expression modules according to their expression trends (Fig. 4C and D), aiming to find a group of metabolites with obvious changes in asthmatic mice that would be restored by AZM administration. Finally, metabolites in cluster 5 in positive ion mode and cluster 6 in negative ion mode were selected for further analysis, and these metabolites are listed in Table S1. These metabolite modules mainly focused on sphingomyelin (SM) metabolism, short-chain fatty acid and unsaturated fatty acid metabolism, energy metabolism, various amino acid metabolism, nucleoside metabolism, and other metabolic pathways. Further differential analysis identified that several cluster 5 metabolites, including phosphorylcholine, phytosphingosine involved in SM metabolism, 2-hydroxy-4-methylbenzoic acid, 7,8-dihydrobiopterin, Met-Met-Arg, and Met-Pro-Arg were significantly decreased in HDM asthmatic mice and upregulated via AZM treatment (Fig. S5). In addition, for metabolites in cluster 6, 7-(3,4-dihydroxyphenyl)-1- (4-hydroxyphenyl)-heptan-3-yl acetate, dulcitol, and picolinic acid were found to be significantly decreased in asthma and upregulated via AZM treatment (Fig. S6).

In conclusion, our results showed that HDM asthmatic mice were characterized by unique metabolic pattern. Several metabolites, especially for SM metabolism-associated metabolites, were significantly decreased in HDM asthmatic mice and restored via AZM treatment.

### Azithromycin regulates airway microbiota-associated sphingomyelin metabolites

**Fig 5:**
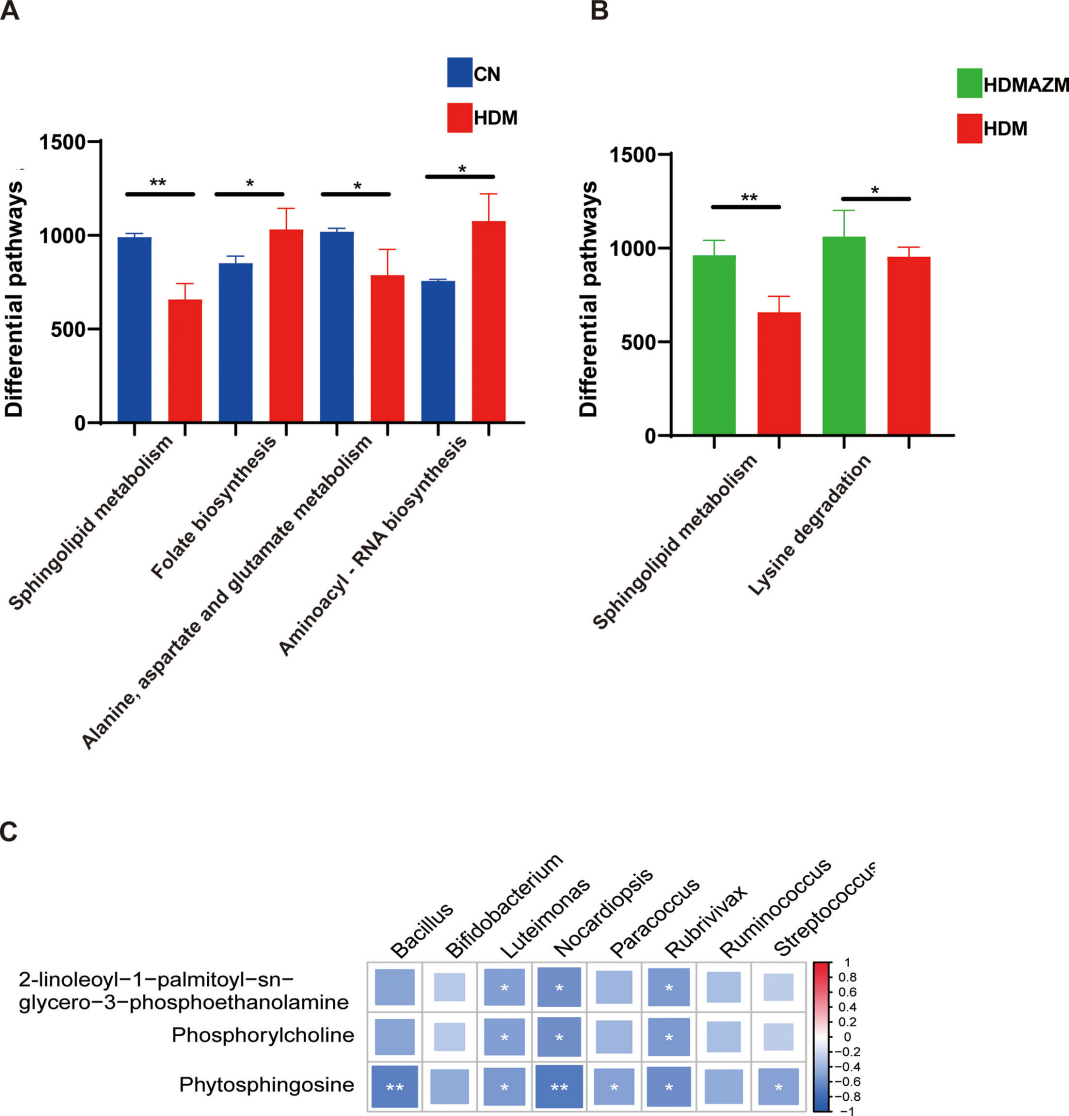
Azithromycin regulates airway microbiota-associated sphingomyelin metabolism. (**A**) Differential KEGG pathways based on PICRUSt2 analysis showed airway microbiota in HDM asthmatic mice had significantly impaired sphingomyelin metabolism. *P < 0.05 and **P < 0.01. (**B**) Differential KEGG pathways based on PICRUSt2 analysis show that azithromycin restores sphingomyelin metabolism of airway microbiota in HDM asthmatic mice. *P < 0.05 and **P < 0.01. (**C**) Spearman analysis showed that metabolites involved in sphingomyelin metabolism were significantly associated with several genera candidates associated with AZM treatment for asthma. *P < 0.05 and **P < 0.01.

The interplay between microbiota and metabolites is common. To identify whether the metabolic change was associated with the alteration of airway microbiota, PICRUSt2 analysis based on KEGG was performed to predict microbiota-associated metabolic pathways. Differential pathway analysis demonstrated that compared with the CN group, airway microbiota in the HDM group had significantly lower activity of SM metabolism (Fig. 5A). Interestingly, AZM administration would significantly recover the activity of SM metabolism (Fig. 5B), suggesting that AZM could regulate microbiota-associated SM metabolism and may further influence several metabolites involved in SM metabolism pathway. Importantly, correlation analysis demonstrated that all three SM metabolism-associated metabolites: phosphorylcholine, phytosphingosine, and 2-linoleoyl-1-palmitoyl-sn-glycero-3-phosphoethanolamine were significantly associated with the eight genera candidate associated with AZM treatment for asthma (Fig. 5C).

In summary, AZM might restore the metabolism activity of the airway microbiota involved in SM metabolism, which was notably diminished in HDM-induced asthmatic mice.

### Sphingomyelin alleviates airway inflammation in HDM-induced asthma mice

**Fig 6:**
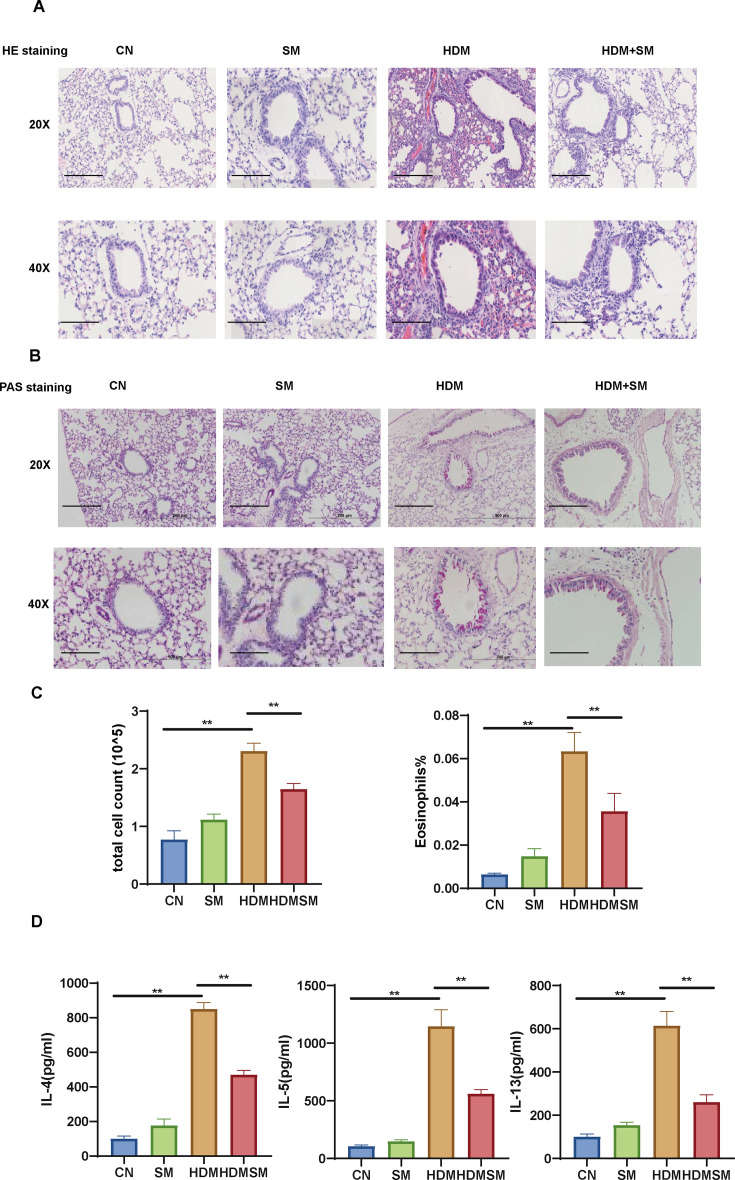
Sphingomyelin alleviates airway inflammation in HDM-induced asthma mice model. (**A**) Representative H & E-stained lung tissue sections from different groups. Magnification, 20× (upper panel, scale bar = 200 µm) and 40× (lower panel, scale bar = 100 µm). (**B**) Representative PAS-stained lung tissue sections from different groups. Magnification, 20× (upper panel, scale bar = 200 µm) and 40× (lower panel, scale bar = 100 µm). (**C**) BALF total cell count and BALF eosinophilia percentage among groups. *P < 0.05 and **P < 0.01. (**D**) BALF IL-4, IL-5, and IL-13 levels among groups. Data are expressed as mean ± standard deviation. *P < 0.05 and **P < 0.01.

The former part of the results suggests that AZM treatment would alter asthma airway microbiota and further affect SM metabolism. To verify the therapeutic efficacy of SM in asthma, we treated HDM asthmatic mice with SM or saline drops daily during the challenge period. HE staining of lung histopathological sections of mice showed that when HDM asthmatic mice were administrated with SM, airway inflammatory cell infiltration was significantly reduced (Fig. 6A). SM treatment alone will not cause significant airway inflammation. In addition, mucus secretions in airway epithelial cells were evaluated in the four groups (Fig. 6B). Similarly, mucus secretion of HDM-induced asthmatic mice was reduced by SM administration. Measurement of BALF inflammatory cell counts (Fig. 6C) and T2 cytokines (Fig. 6D) in four groups was conducted. The results showed that compared with the HDM group, SM administration will alleviate airway inflammation, manifested by lower total cell counts, EOS percentage, and T2 cytokines. In conclusion, SM significantly reduced type 2 airway inflammation and mucus secretion in HDM asthmatic mice.

## DISCUSSION

Our study presents evidence that AZM effectively mitigates type 2 airway inflammation in an HDM-induced asthma model in mice. The therapeutic effect is partially attributed to the drug’s ability to restore airway microbiota imbalances and modulate the metabolism of microbiota-associated SM. SM itself can alleviate airway inflammation in asthmatic mice, which led to a significant reduction in airway inflammatory cell infiltration and mucus secretion, aligning with decreased levels of T2 cytokines, indicative of its potent anti-inflammatory action.

In this study, we concentrated on type 2 inflammation in asthma and discovered that diminished diversity of lung microbiota correlates with elevated levels of IL-5 and eosinophil percentage (EOS%). Recent research has underscored the pivotal role of lung microbiota in the development and treatment of asthma, especially concerning type 2 inflammation (10). Experiments with germ-free mice, which are deprived of microbial exposure, demonstrate more severe type 2 airway inflammation compared to mice with a normal microbiota. Furthermore, the increased allergic inflammation in germ-free mice can be diminished to levels seen in specific pathogen-free (SPF) mice when germ-free mice are co-housed with SPF mice for 3 weeks (31). Patients with type 2-high asthma, characterized by eosinophilic inflammation, exhibit a specific alteration of airway bacteria. Durack et al. (32) observed that sputum bacterial load is inversely related to the bronchial expression of type 2-related genes. An earlier study identified that sputum *Streptococcal* OTUs were linked to recent-onset asthma, rhinosinusitis, and sputum eosinophilia (33). Additionally, a study involving 167 participants found that eosinophilic asthma was associated with the increased relative abundance of *Streptococcus*, *Gemella*, and *Porphyromonas* taxa (34).

The exact mechanisms through which azithromycin exerts its effects in asthma with type 2 inflammation are not fully understood. A previous study showed that the effect of AZM on treating asthma-like symptoms in preschool children was associated with microbiota richness in hypopharyngeal aspirates (35). Another study found that AZM treatment in severe asthma induced a significant decrease in the total diversity of taxa, including a significant decline in the pathogenic species *H. influenzae* (24). These two separate studies have indicated that the therapeutic effects of AZM treatment could, to some extent, be attributed to its antimicrobial function. It is crucially important to highlight that our research has contributed further evidence to substantiate the idea that the administration of AZM holds the potential to partially correct the dysbiosis in the airway microbiota, bringing it back to a more normal and healthy state. In addition, the reduction of *Streptococcus*, *Ruminococcus*, *Bifidobacterium*, *Paracoccus*, *Rubrivivax*, *Luteimonas*, *Bacillus,* and *Nocardiopsis* was found to be associated with AZM treatment effect. Some members of these bacterial genera had previously been recognized as having a significant association with asthma. For example, a recent study showed that sputum microbiota in severe asthma differs from healthy controls and non-severe asthmatics. *Streptococcal* OTUs were associated with recent-onset asthma and type 2 inflammation, including sputum eosinophilia (33). *Bacillus* species, including *Bacillus subtilis*, have been identified in the microbiota of asthma patients (36). *Bacillus subtilis* enzymes have been linked to allergic reactions in the lungs, indicating a potential role in asthma pathogenesis (37).

Previous studies have reported that the metabolites of the microbial microbiota in the host can regulate the disease and health of the host (38). PICRUSt2 analysis demonstrated that the HDM group had significantly lower activity of SM metabolism and AZM administration would significantly recover the activity of SM metabolism. Importantly, through metabolomics analysis, we found that phosphorylcholine and phytosphingosine, which are involved in SM metabolism, were downregulated in the HDM asthmatic mice. This was associated with several genus candidates associated with AZM treatment for asthma. We further validated and found that SM drop nasal treatment decreased the number of total cells and Th2 cytokines in BALF, alleviated airway and perivascular type 2 inflammation, and reduced airway mucus secretion in HDM-induced asthmatic mice. SM is a phospholipid that is mainly found in invertebrates, accounting for 51% of the total phospholipids (39). It has a significant impact on cell proliferation, apoptosis, and survival, which, functioning as transmembrane proteins and signal transduction molecules, contribute to preserving the overall health state of the body (39). With the rise of sequencing technology, SM began to be regarded as a microbiota metabolite. An animal study found that dietary intake of specific lipids (e.g., phosphatidylcholine and SM) has significant reduction effects on inflammatory gut disease damage and pro-inflammatory cytokines in IBD, and this effect is correlated with *Lactobacillus* and *Faecium* in the gut (40). In addition, a clinical trial found that microbiota-associated metabolites, such as SM, cooperate to regulate adult blood pressure (41). The findings suggest that SM, a bacterial metabolite, is involved in several disease progression. As for asthma, SM has been implicated in the pathogenesis of asthma with type 2 inflammation. A recent study found that lower levels of SM in early childhood are associated with an increased risk of developing asthma before age 3 and increased airway resistance at age 6, most of them are with type 2 inflammation (42). Another study reported that asthmatic patients show significantly decreased levels of specific SM, compared to healthy controls (43). In our study, we demonstrated that the AZM treatment effect might be associated with its regulation of SM metabolism ability of airway microbiota.

In summary, our study reveals that AZM can alleviate type 2 airway inflammation in a murine model of asthma by regulating the airway microbiota and its associated SM metabolism. This research highlights the potential for microbiota-targeted therapies in asthma and underscores the need for further investigation into the clinical applicability of these findings. Our study offers valuable insights, yet it is not without limitations. Both past and current research indicate that the airway microbiome exhibits low diversity and bacterial load, with considerable variability in the airway microbiota (44). Consequently, uncontrollable changes in the airway microbiota may occur during the induction of an asthmatic model and drug intervention, leading to some inevitable discrepancies. Currently, there are no reliable and scientific methods to effectively transplant the airway microbiota, and thus no direct means to verify its effects on AZM treatment. Second, our study only used HDM asthmatic animal models, which may limit the extrapolation of our results. We suggest that future studies employ larger sample sizes and diverse models to validate our findings in different phenotypes of asthma. Additionally, while we found that AZM can regulate metabolites associated with the airway microbiota, our study did not directly demonstrate the specific mechanisms by which these metabolites act in the context of AZM treatment for asthma. Fourth, our study was conducted in animal models, and its clinical relevance and efficacy need to be further confirmed through clinical trials. Finally, our study did not assess the long-term effects of AZM on the microbiota and metabolites, which is an important aspect for future research to consider.

### Conclusion

The present study provides evidence that AZM effectively mitigates type 2 airway inflammation in an HDM-induced asthmatic mouse model. The study demonstrates that AZM’s therapeutic effects are associated with its capacity to regulate the airway microbiota and its metabolites, particularly SM metabolism, thereby restoring microbial diversity and reducing airway inflammation. Specifically, AZM was shown to significantly regulate specific genera related to the airway microbiota, such as *Streptococcus*, *Staphylococcus*, *Ruminococcus*, *Coprococcus*, *Bifidobacterium*, and others, and restore microbiota diversity, which in turn alleviates airway inflammation in HDM-induced asthmatic mice. Furthermore, AZM significantly regulated SM metabolism associated with the airway microbiota, and changes in this metabolic pathway were closely correlated with the alleviation of airway type 2 inflammation in asthmatic mice. Our study suggests that the potential of microbiota-targeted therapies, particularly utilizing AZM or its regulated metabolites like SM, should be considered in future asthma treatments. Future research should further explore the long-term impact of AZM on the microbiota and how these changes interact with the pathophysiology of asthma. Additionally, clinical trials are needed to validate the effectiveness and safety of AZM in regulating the microbiota and metabolites in human asthma treatment.

## Data Availability

The original data of 16S rRNA and metabolomics supporting the findings of this work have been uploaded in FigShare, DOI:10.6084/m9.figshare.28334270.

## AFTER EPUB

*[This article was published on 11 February 2025 with errors in the first author's name. The name was corrected in the current version, posted on 19 February 2025.]*

## SUPPLEMENTAL MATERIAL

Supplemental material spectrum.02217-24-s0001.docx Table S1; Fig. S1 to S6.

ASM does not own the copyrights to Supplemental Material that may be linked to, or accessed through, an article. The authors have granted ASM a non-exclusive, world-wide license to publish the Supplemental Material files. Please contact the corresponding author directly for reuse.
