# Supplementary material for: Effects of azithromycin on alleviating airway inflammation in asthmatic mice by regulating airway microbiota and metabolites
Source: Microbiol Spectr. 2025 Feb 11;13(3):e02217-24. doi: 10.1128/spectrum.02217-24 (PMC11878009; doi:10.1128/spectrum.02217-24)
Supplement: Supplemental material — Table S1; Fig. S1 to S6. [file spectrum.02217-24-s0001.docx]

**Supporting Information**

**Effects of** **azithromycin on alleviating airway inflammation in asthmatic mice by regulating airway microbiota and metabolites**

Huang et al.

1. **Supplementary Table S1**
2. **Supplementary Figure S1-S6**
3. **Supplementary Table**

**Table S1 metabolites in cluster 5 of positive ion mode and cluster 6 of negative ion mode and their associated metabolic pathways.**

| **metabolites** | **metabolic pathways** |
| --- | --- |
| Phosphorylcholine | sphingomyelin metabolism |
| 2-linoleoyl-1-palmitoyl-sn-glycero-3-phosphoethanolamine |  |
| Phytosphingosine |  |
| 1-stearoyl-rac-glycerol | Lipid metabolism |
| 15(s)-15-methylprostaglandin f2. alpha.ethylamide |  |
| 4,8-dimethylquinolin-2-ol | Tryptophan metabolism |
| Formylanthranilic acid |  |
| Picolinic acid |  |
| 2-hydroxy-4-methylbenzoic acid |  |
| N6-methyladenine | Nucleotide metabolism |
| 5-methylcytosine |  |
| 7,8-dihydrobiopterin |  |
| Cytisine |  |
| [7-(3,4-dihydroxyphenyl)-1-(4-hydroxyphenyl)heptan-3-yl] acetate | Energy metabolism |
| whileanoic acid |  |
| 1,4-whileynediol |  |
| Dulcitol |  |
| 4-hydroxyphenylacetic acid | Amino acid metabolism |
| 4-imidazoleacrylic acid |  |
| 1-methyl-l-histidine |  |
| Acetylglycine |  |
| DL-cysteine |  |
| Met-Met-Arg |  |
| Met-Pro-Arg |  |
| 3-hydroxy-3-methylglutaric acid |  |
| 4-(2-methyl-4-chlorophenoxy)whileyric acid methyl ester |  |
| 4-hydroxyphenylacetic acid |  |

1. **Supplementary Figures**

**
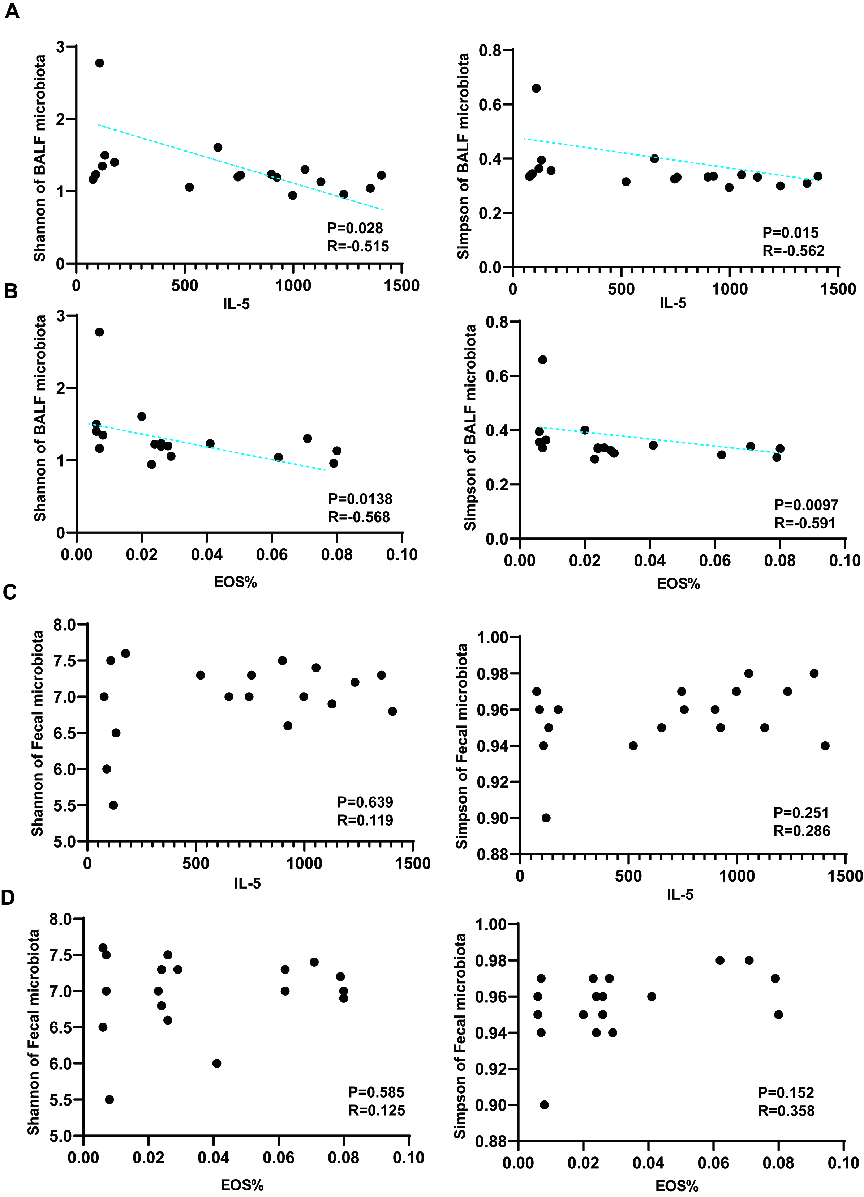
**

**Supplementary figure 1: BALF IL-5 level and EOS% were significantly associated with airway microbiota diversity, rather than intestinal microbiota.**

1. BALF IL-5 level was significantly associated with airway microbiota diversity index.
2. BALF EOS% was significantly associated with airway microbiota diversity index.
3. BALF IL-5 level was not significantly associated with intestinal microbiota diversity index.
4. BALF EOS% was not significantly associated with intestinal microbiota diversity index.

**
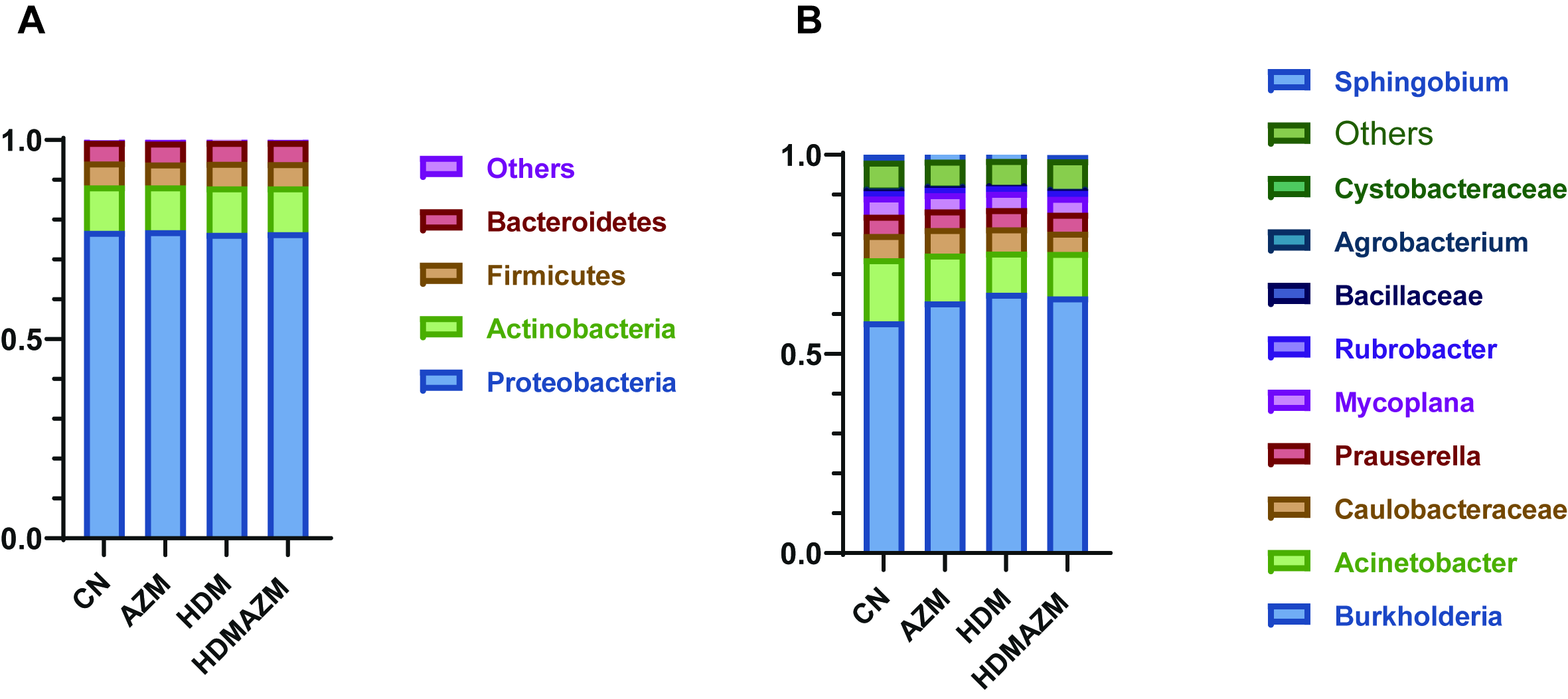
**

**Supplementary figure 2: Taxonomy composition of airway microbiota among groups.**

1. top 4 phyla among groups.
2. Top 10 genera among groups.

**
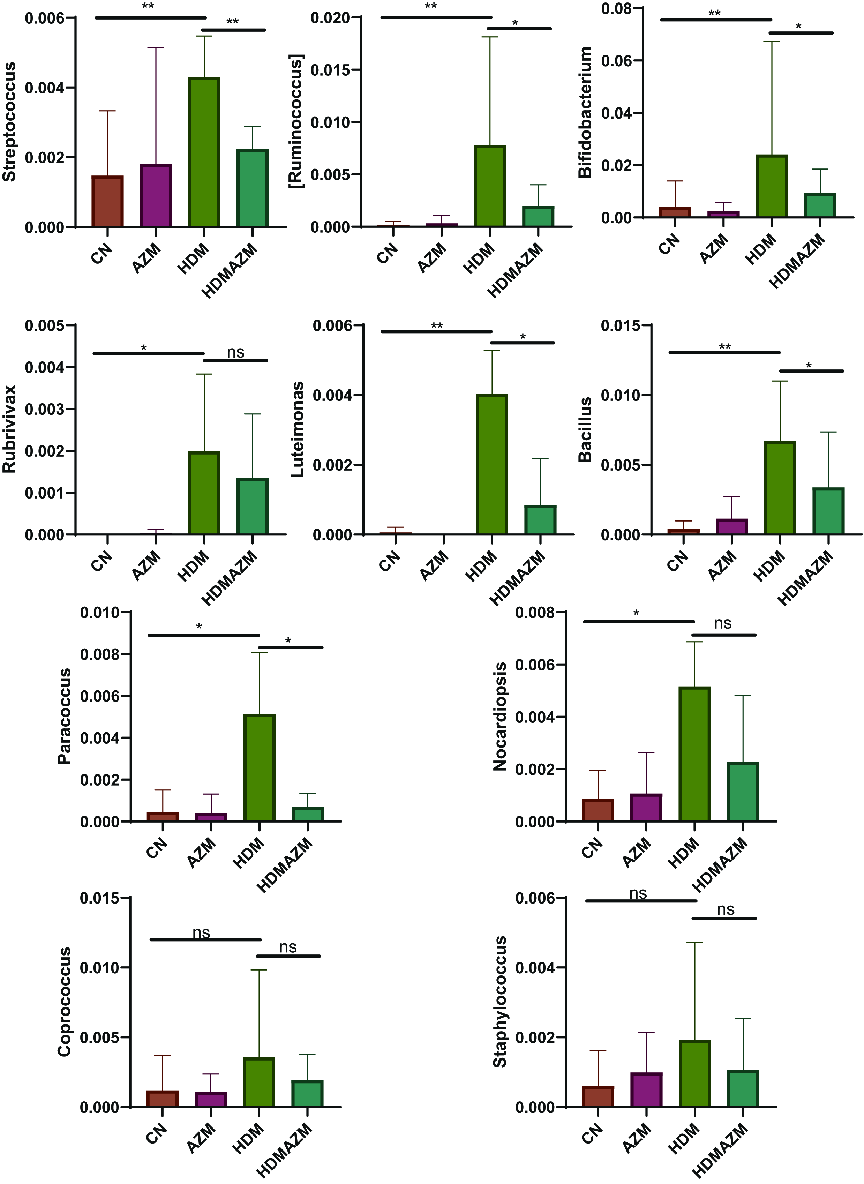
**

**Supplementary figure 3: Distribution trend of potential genera candidate among groups. Mann-Whitney U test was used to calculate statistical significance (P-value). * p < 0.05, ** p < 0.01. ns:non-significant.**

**
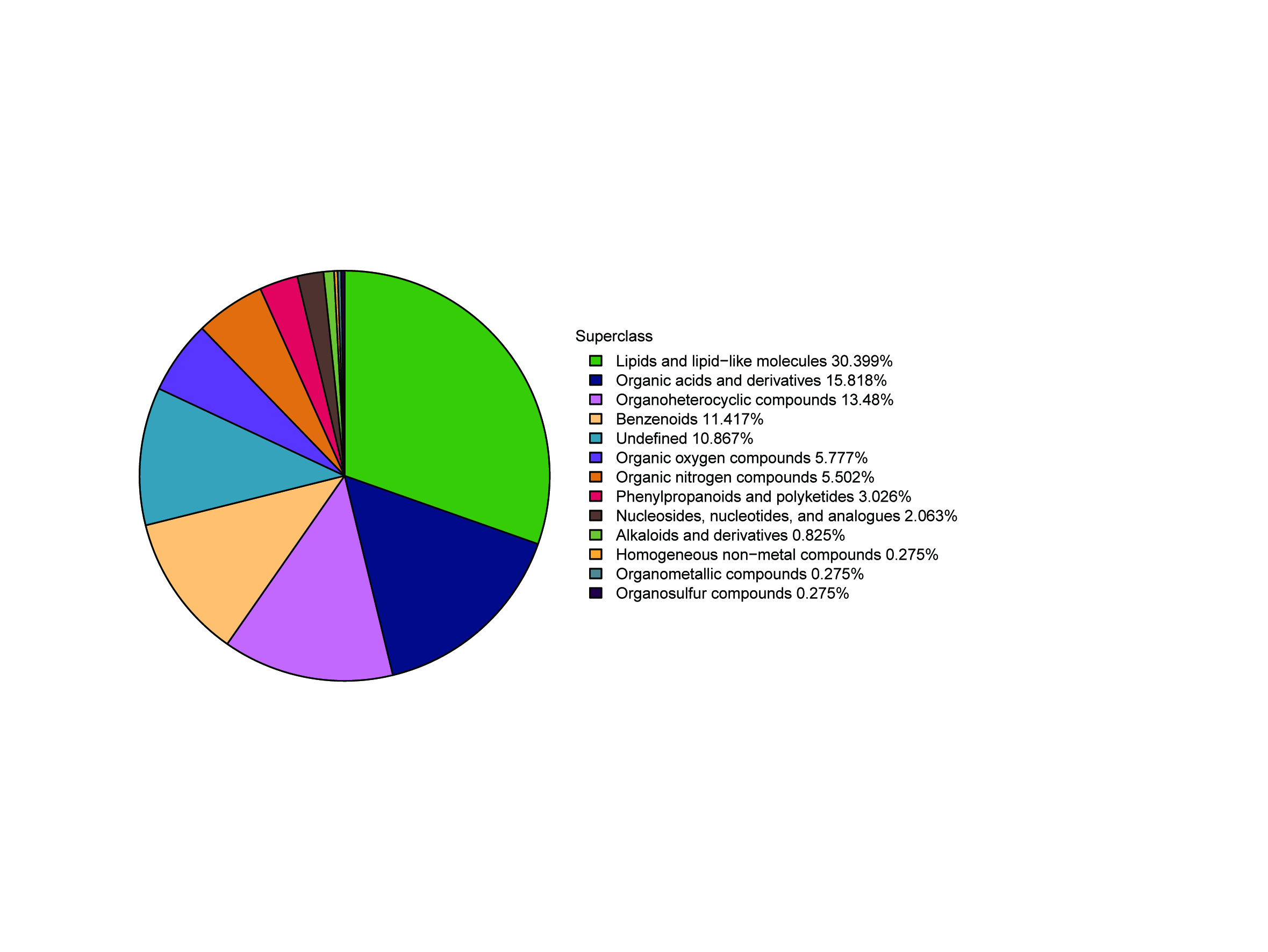
**

**Supplementary figure 4: pie chart shows the proportion of all metabolites in both positive and negtive ione mode identified by LC-MS/MS.**

**
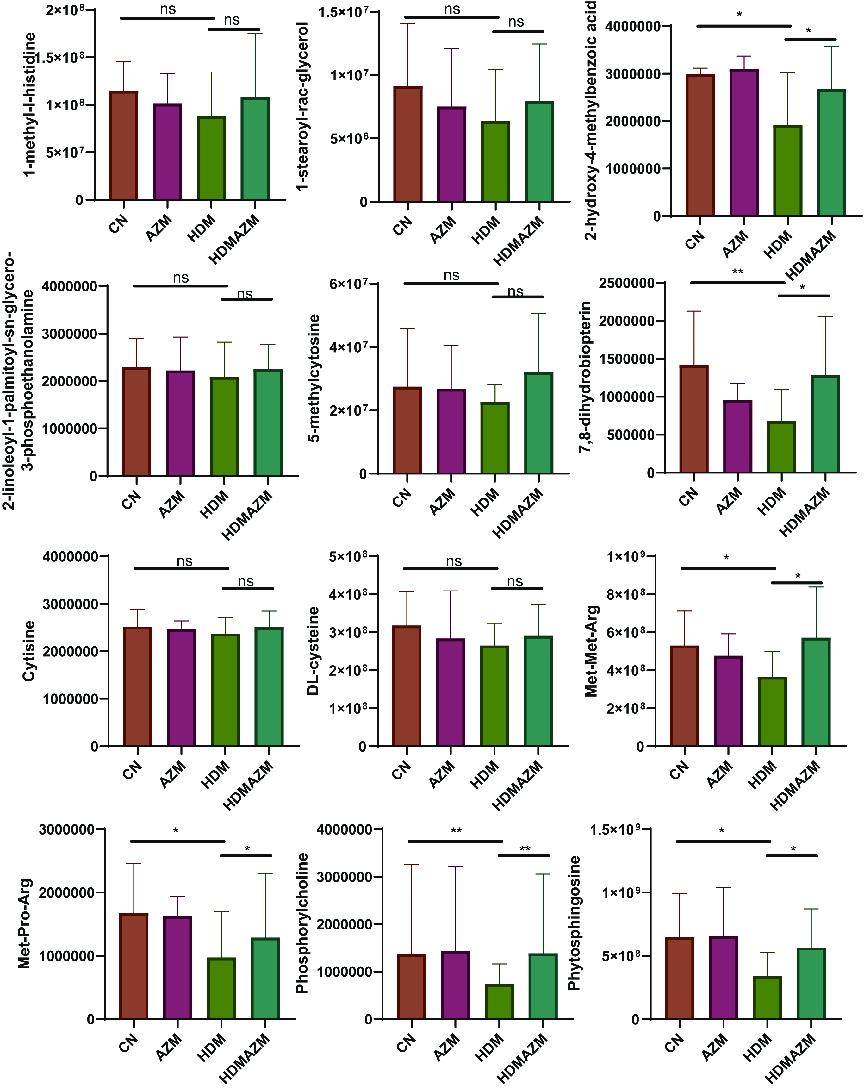
**

**Supplementary figure 5: Distribution trend of potential metabolites candidate in positive ion mode among groups. Mann-Whitney U test was used to calculate statistical significance (P-value). * p < 0.05, ** p < 0.01. ns:non-significant.**

**
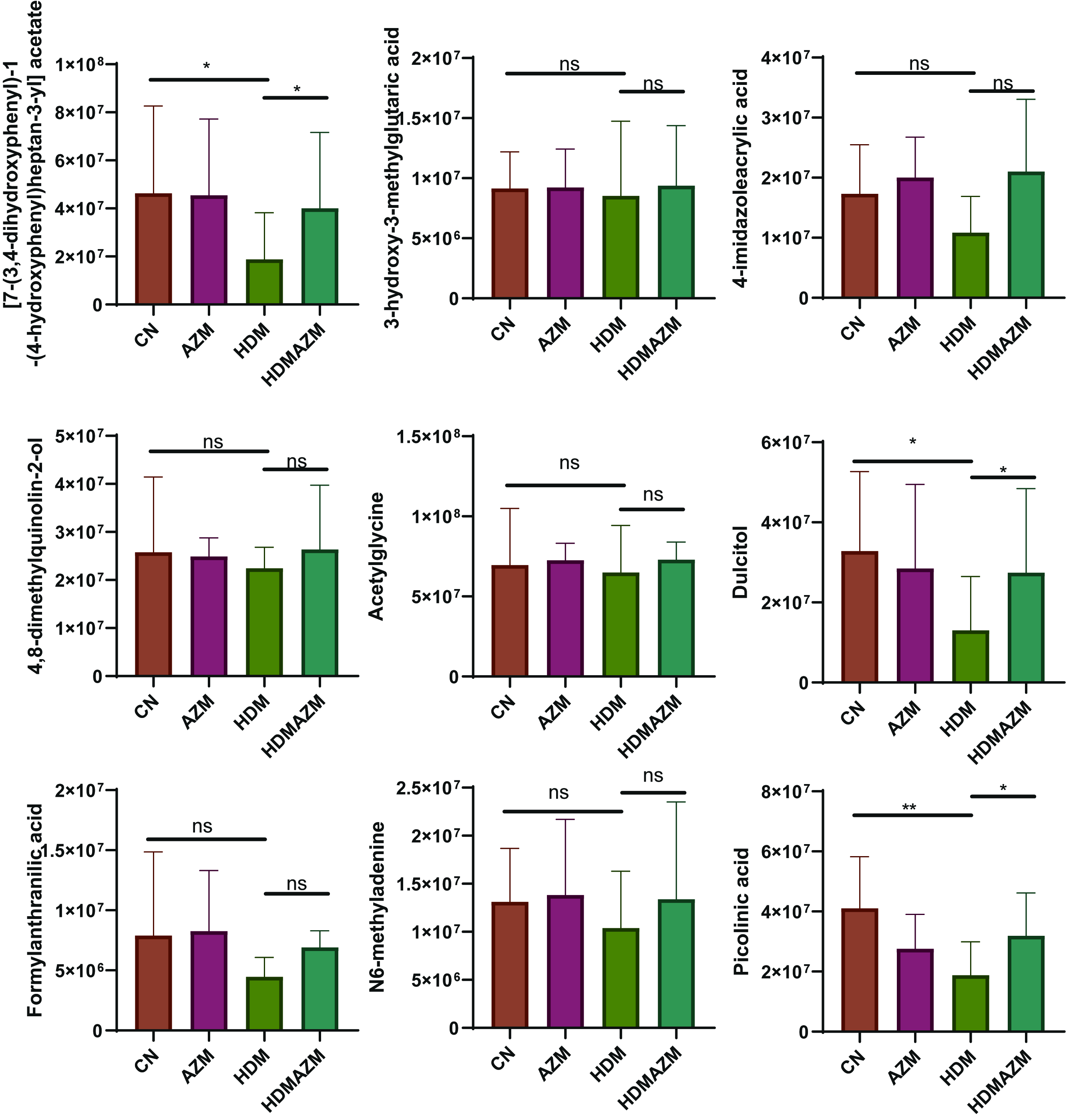
**

**Supplementary figure 6: Distribution trend of potential metabolites candidate in negative ion mode among groups. Mann-Whitney U test was used to calculate statistical significance (P-value). * p < 0.05, ** p < 0.01. ns:non-significant.**

1. **Supplementary materials and methods**

**Statistical analysis**

The continuous variables were compared between two independent groups by Mann–Whitney U test or independent t-test. Wilcoxon signed-rank test is used for paired sample testing. The categorical variables were compared by Fisher’s exact test. p-values < 0.05 were considered statistically significant. P-value <0.05 was considered statistically significant. Spearman’s analysis was used to explore the correlation between different taxonomies and between taxonomies and metabolites.

**IHC**

The IHC was carried out as previously described. The Antigen retrieval was conducted by pressure cooker at full pressure for 3min in the EDTA Buffer pH=8.0 (ORIGENE # ZLI-9067) and then blocked in 10% FBS for 1 hour. Then the paraffin sections were incubated with the first antibodies diluted in antibodies diluent buffer (ORIGENE # ZLI-9029) at 4℃ overnight and with Horseradish Peroxidase (HRP)-conjugated secondary antibody (ORIGENE # ZLI-9017) at 37℃ for 1h. Finally, the DAB chromogenic solutions (ORIGENE # ZLI-9017) were used to detect the [positive](file:///C:/Program%2520Files%2520(x86)/Youdao/Dict/8.9.3.0/resultui/html/index.html#/javascript:;) [staining](file:///C:/Program%2520Files%2520(x86)/Youdao/Dict/8.9.3.0/resultui/html/index.html#/javascript:;).

**Human Lung caner tissue Analysis**

70 tissues were collected from patients with lung cancer receiving immunotherapy, who were subjected to surgery form Jan. 2010 to Jun. 2020 at Nangfang hospital, Southern Medical University, China. These samples were evaluated by a experienced pathologist. All of the patients had received at least one immunotherapy. These samples were detected to estimate oil-red expression. This research was approved by the Research Ethics Committee of the Nangfang hospital. The written informed consent was obtained from all the patients.

**Cell culture , Nutrients starvation**

A549 and H1650, human lung carcinoma cell lines were preserved in the Nangfang hospital, and cultured in RPMI1640 (Invitrogen #C11875500BT) supplemented with 10% FBS (Invitrogen #10099-141), 100 units/ml penicillin, and 100 μg/ml streptomycin in a humidified atmosphere containing 5% CO_2_. All cell lines were authenticated by STR DNA profiling (Microread Diagnostics Co., Ltd, Guangzhou, China). Nutrients starvation of cells was first cultured in total nutrient medium with 10% FBS for at least 3 days, followed by 24h starvation of 1%FBS or glucose using different nutrient deficient mediums (Chenxue Biotech #CM10043).

### **T cell isolation**

All cytokines were purchased from Peprotech (Rocky Hill, NJ, USA). Human blood was obtained from healthy volunteers after informed consent and with approval by the Nanfang hospital uman Participant Ethics Committee (Ethics Approval 010558). CD3+ T cells were isolated by negative selection from freshly obtained peripheral blood mononuclear cells using human CD3+ T cell isolation kit (ImunoSep,Cat#710205)

CD8+ T cells were isolated by negative selection from freshly obtained peripheral blood mononuclear cells using human CD8+ T cell isolation kit (ImunoSep,Cat#710805). Purity was confirmed with CD3E-FITC (BD Biosciences, Cat# 561806) and CD8A-FITC (Cat# CST, Cat# [85336](https://www.cellsignal.cn/products/primary-antibodies/cd8a-d8a8y-rabbit-mab/85336) ) and analyzed by flow cytometry. Cells were cultured in RPMI 1640 medium (Sigma) supplemented with 10% heat-inactivated endotoxin-tested FCS (Biochrom GmbH, Berlin, Germany).

### **T cell migration assay**

human T cell migration was investigated by trans-well migration assays (Corning). 106 transduced T cells were placed into the upper chamber of a trans-well plate with a 3 μm pore filter. The lower chamber contained different tumour cell supernatant. To generate tumour cell supernatant, 105 A549 orH1650 lung carcinoma cells were seeded into 6-well plates and starvation for FBS or glucose for 24 h and supernatants were harvested and used for migration assay. The numbers of migrated cells in the lower chamber were quantified by FACS analysis after an incubation at 37°C for 3 – 4 hours.

### **Antibodies and flow cytometry**

The surface antigens CD8, CD3E, and IFN gamma were stained with the following fluorescent labeled antibodies: anti CD8A (CST, Cat# [85336](https://www.cellsignal.cn/products/primary-antibodies/cd8a-d8a8y-rabbit-mab/85336) ), anti-CD3E (BD Biosciences, Cat# 561806) and anti IFN gamma (CST, Cat# 8455) for 30 min at 4 °C. Cells were fixed in 1% paraformaldehyde and analyzed by flow cytometry (FACS Canto II, BD Biosciences). The antibodies:CD8a (CST, Cat# [85336](https://www.cellsignal.cn/products/primary-antibodies/cd8a-d8a8y-rabbit-mab/85336) ), PD-L1(CST, Cat# [86163](https://www.cellsignal.cn/products/primary-antibodies/cd8a-d8a8y-rabbit-mab/85336) ), GAPDH(CST, Cat# [2118](https://www.cellsignal.cn/products/primary-antibodies/cd8a-d8a8y-rabbit-mab/85336) ) was used for IHC or Western blot experiments.
